# New Genus, a New Species, and New Combinations in Edessinae (Hemiptera: Pentatomidae)

**DOI:** 10.1007/s13744-026-01425-3

**Published:** 2026-08-24

**Authors:** Eduardo Victor de Paiva Cunha, Talita Naiff Robert, Mayara Santos Gomes, José Antônio Marin Fernandes

**Affiliations:** 1 Programa de Pós-Graduação em Zoologia, Instituto de Ciências Biológicas, Universidade Federal do Pará, Belém, Pará Brazil; 2 Instituto de Ciências Biológicas, Laboratório de Invertebrados (LA-INV), Universidade Federal do Pará, Belém, Pará Brazil

**Keywords:** *Notochlora*, Taxonomy, Morphology, Edessinae, Amazon rainforest, Atlantic Forest

## Abstract

A new genus of Edessinae is described, *Notochlora* gen. nov. Cunha, Robert and Fernandes. The new genus comprises three species, including two species transferred from *Edessa* and one new species: *Notochlora dolichocera* (Lichtenstein 1795) comb. nov., *N. guyanensis* (Fernandes and van Doesburg 2000) comb. nov., and *Notochlora chlorosa* Cunha, Robert and Fernandes sp. nov. These species are distributed in Suriname, Guyana, French Guiana, Brazil, Ecuador, Peru, and Bolivia. Diagnoses, redescriptions, illustrations, and a distribution map for the species of *Notochlora* gen. nov. are provided. Additionally, *Edessa nigromaculata* Fernandes and van Doesburg 2000 is transferred to *Grammedessa* Correa and Fernandes, 2016, based on morphological evidence.

## Introduction

The genus *Edessa* Fabricius (1803) has been considered problematic from a taxonomic point of view (Santos et al. 2015; Fernandes et al. 2018; Nunes et al. 2020), morphologically diverse, and non-monophyletic (Nunes et al. 2019, 2020; Da Silva and Fernandes 2021 ). Currently, *Edessa* comprises three subgenera (*Edessa*, *Aceratodes*, and *Dorypleura*). The nominal subgenus was recently revised and redefined by Mendonça et al. (2023), representing an important step toward clarifying the internal organization of the genus. In addition, *Edessa* has 11 species groups that have been proposed to partially organize its diversity, but only five show diagnostic features that link them to one of the subgenera: the ***E. rufomarginata*** group is assigned to *Edessa* (*Aceratodes*) (Silva et al. 2006), whereas the ***E. sexdens***, ***E. ovina***, ***E. metallica***, and ***E. cervus*** groups belong to the nominal subgenus (Mendonça et al. 2023).

One of the species groups of *Edessa* is *E. dolichocera* group, created by Fernandes and van Doesburg (2000) to include three species: *E. dolichocera* (Lichtenstein 1795), *E. guyanensis* Fernandes and van Doesburg 2000, and *E. nigromaculata* Fernandes and van Doesburg 2000. However, recent analyses indicate that *E. nigromaculata* belongs to the genus *Grammedessa* Correa and Fernandes, 2016 . More recently, a new species closely related to *E. dolichocera* and *E. guyanensis* has been recognized. This group exhibits morphological traits that clearly distinguish it from *Edessa*.

In this context, the present study aims to (i) describe a new genus within Edessinae; (ii) redescribe three species; (iii) describe a new species for the new genus; and (iv) transfer species of *Edessa* to other taxa, namely two species to the new genus and one species to *Grammedessa*.

## Material and Methods

We examined 56 specimens belonging to the collections mentioned below: ESALQ – Escola Superior de Agricultura “Luiz de Queiroz,” Piracicaba, São Paulo, Brazil; FURG – Universidade Federal do Rio Grande do Sul, Porto Alegre, Brazil; MNRJ – Museu Nacional do Rio de Janeiro, Rio de Janeiro, Brazil; MZSP – Museu de Zoologia da Universidade de São Paulo, São Paulo, Brazil; NNH – Nationaal Natuurhistorische Museum, Leiden, Netherlands; RL – Roland Lupoli Collection, Fontenay-sous-Bois, France; UFPA – Universidade Federal do Pará, Belém, Pará, Brazil.

The terminology used for species descriptions follows that of Fernandes (2010), the scent gland apparatus of Kment and Vilímová (2010), for male genitalia Dupuis (1970), and for female genitalia Zhou and Redei (2020). The male genitalia cleaning and removal technique was described in Mendonça et al (2021). Images were captured using the Leica DFC 450 camera, connected to the Leica M205A stereomicroscope, and then stacked with Helicon Focus. All the measurements expressing the size of the specimens were carried out using a micrometric eyepiece coupled to the eyepiece of the Zeiss Discovery V8 stereomicroscope and are presented in millimeters. Maps were made using QGIS (2025).

## Results

**Fig. 1:**
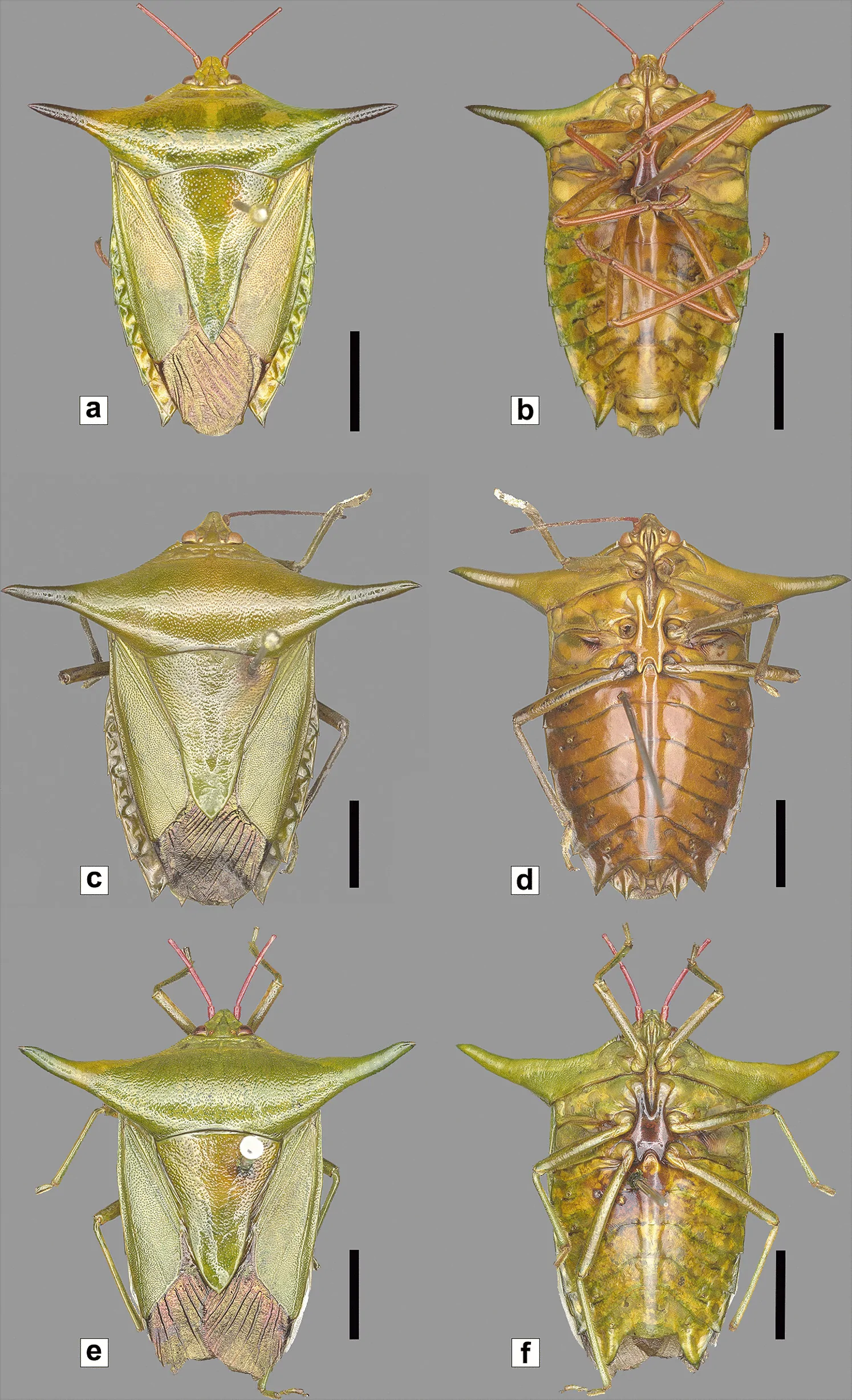
Dorsal and ventral view. **A**, **B**—*Notochlora dolichocera*; **C**, **D**—*Notochlora guyanensis*; **E**, **F**—*Notochlora chlorosa*. Scale bars = 5 mm

**Fig. 2:**
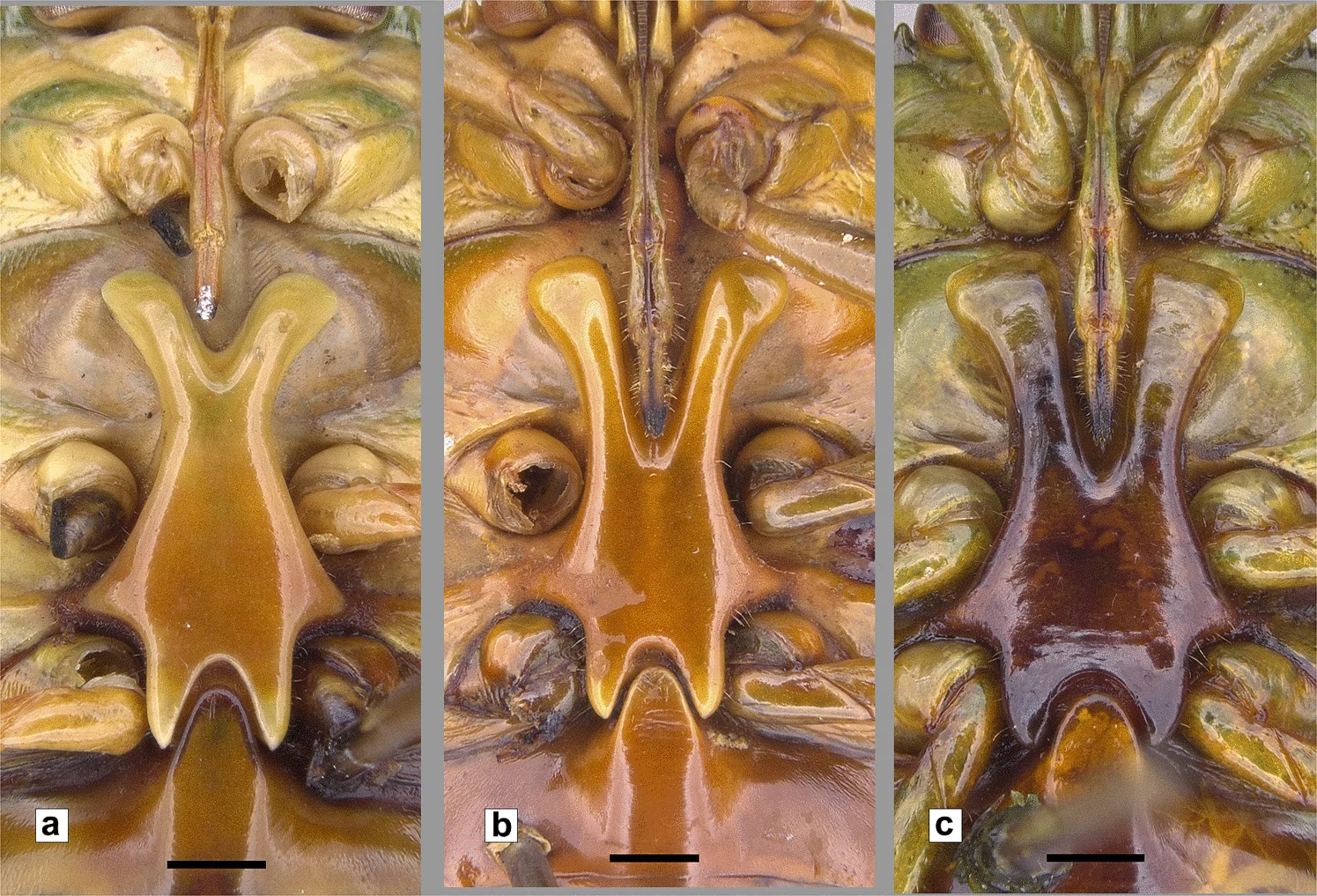
Metasternal process: **A**—*Notochlora dolichocera* comb. nov., **B**—*Notochlora guyanensis* comb. nov., **C**—*Notochlora chlorosa* sp. nov. Scale bars = 5 mm

**Fig. 3:**
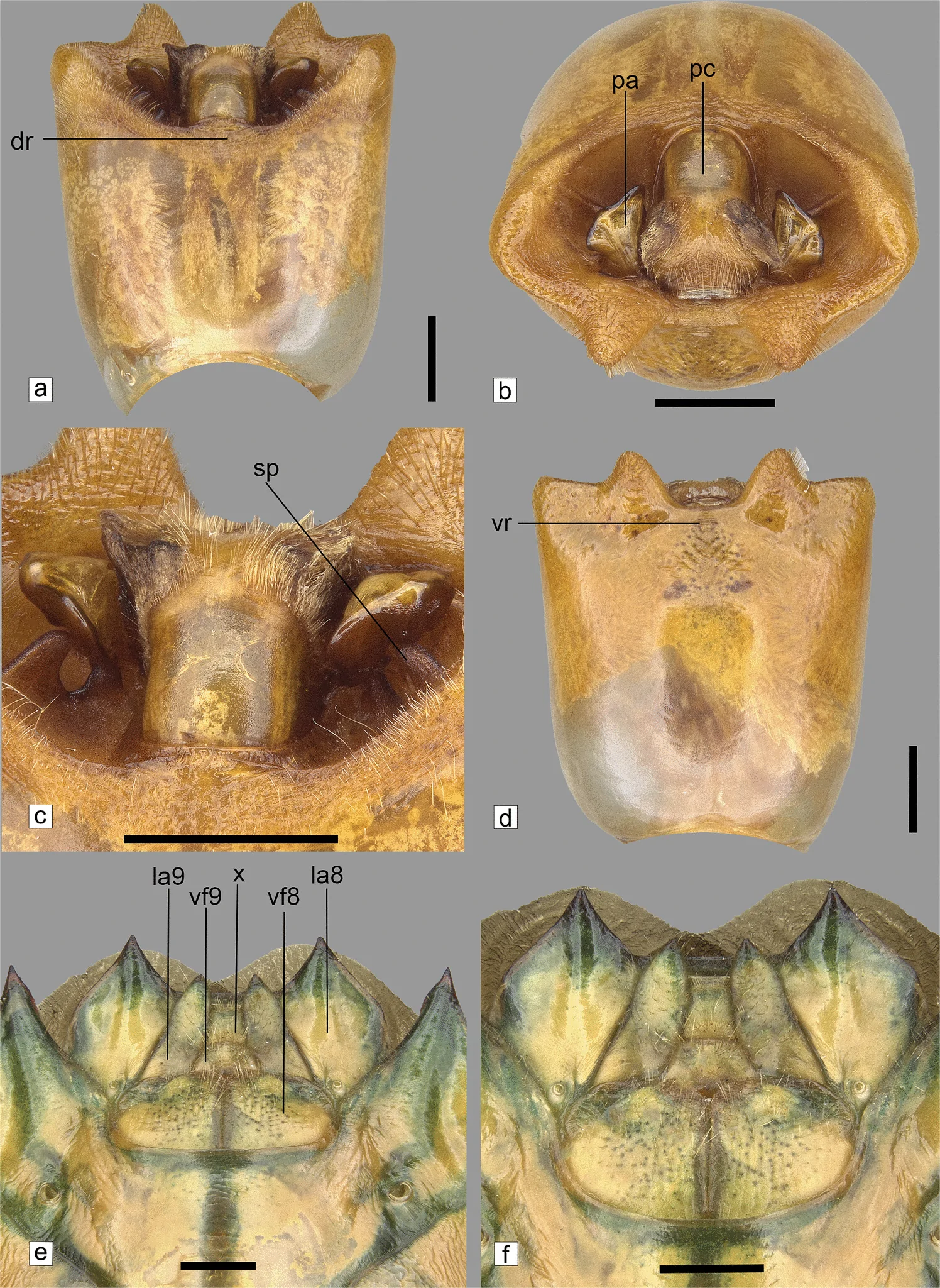
*Notochlora dolichocera* comb. nov. male genitalia (pygophore): **A**—dorsal view, **B**—posterior view, **C**—dorso-posterior view, **D**—ventral view. Female genitalia: **E**—ventral view, **F**—ventro-posterior view. Acronyms: Dr—dorsal rim; Sp—superior process of the genital cup; Pa—paramere; Pc—proctiger; Vr—ventral rim; Vf8—valvifers VIII; Vf9—valvifers IX; La8—laterotergites VIII; La9—laterotergites IX; X—tenth segment. Scale bars = 1 mm

**Fig. 4:**
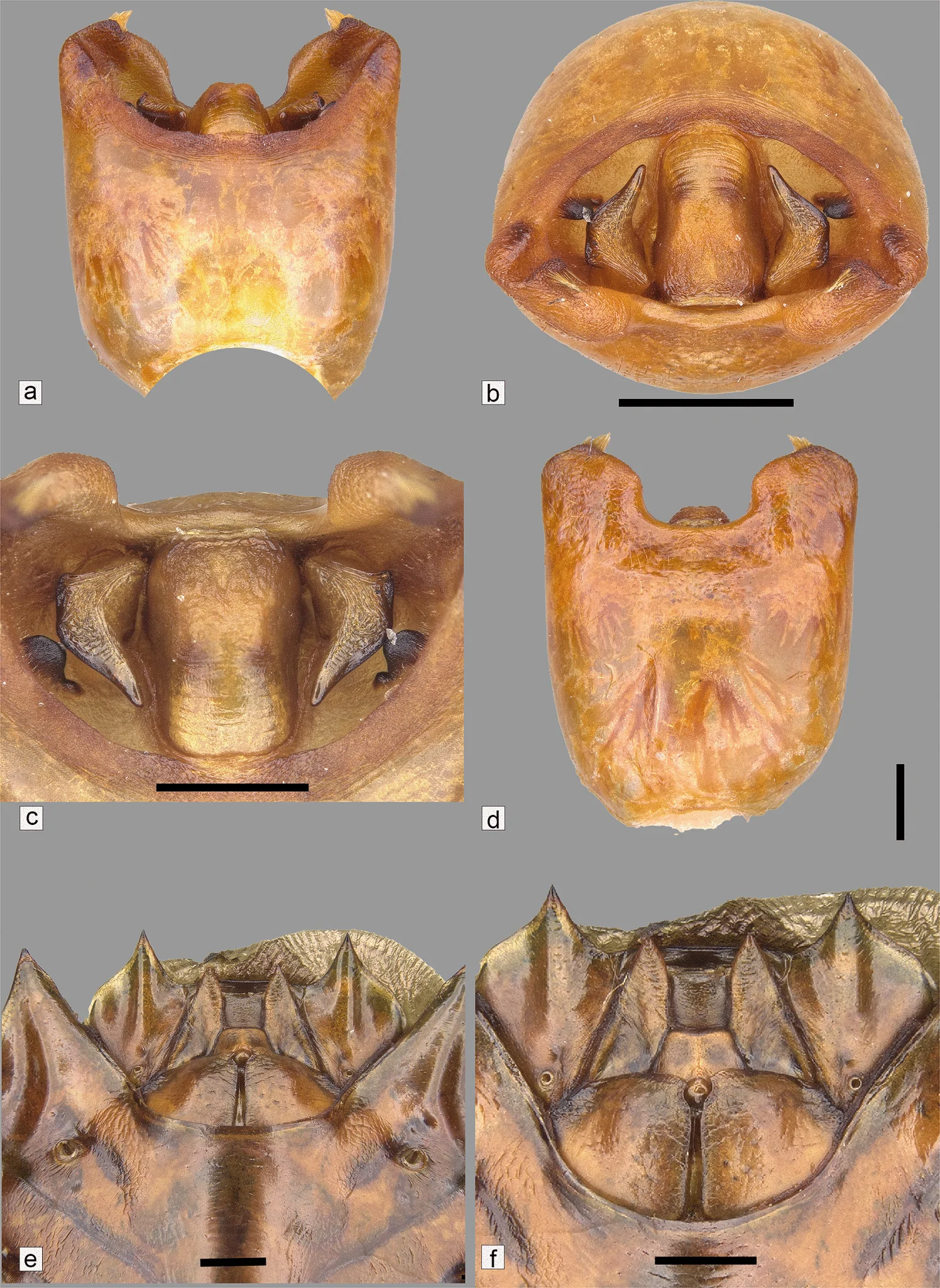
*Notochlora guyanensis* comb. nov. male genitalia (pygophore): **A**—dorsal view, **B**—posterior view, **C**—dorso-posterior view, **D**—ventral view. Female genitalia: **E**—ventral view, **F**—ventro-posterior view. Scale bars = 1 mm

**Fig. 5:**
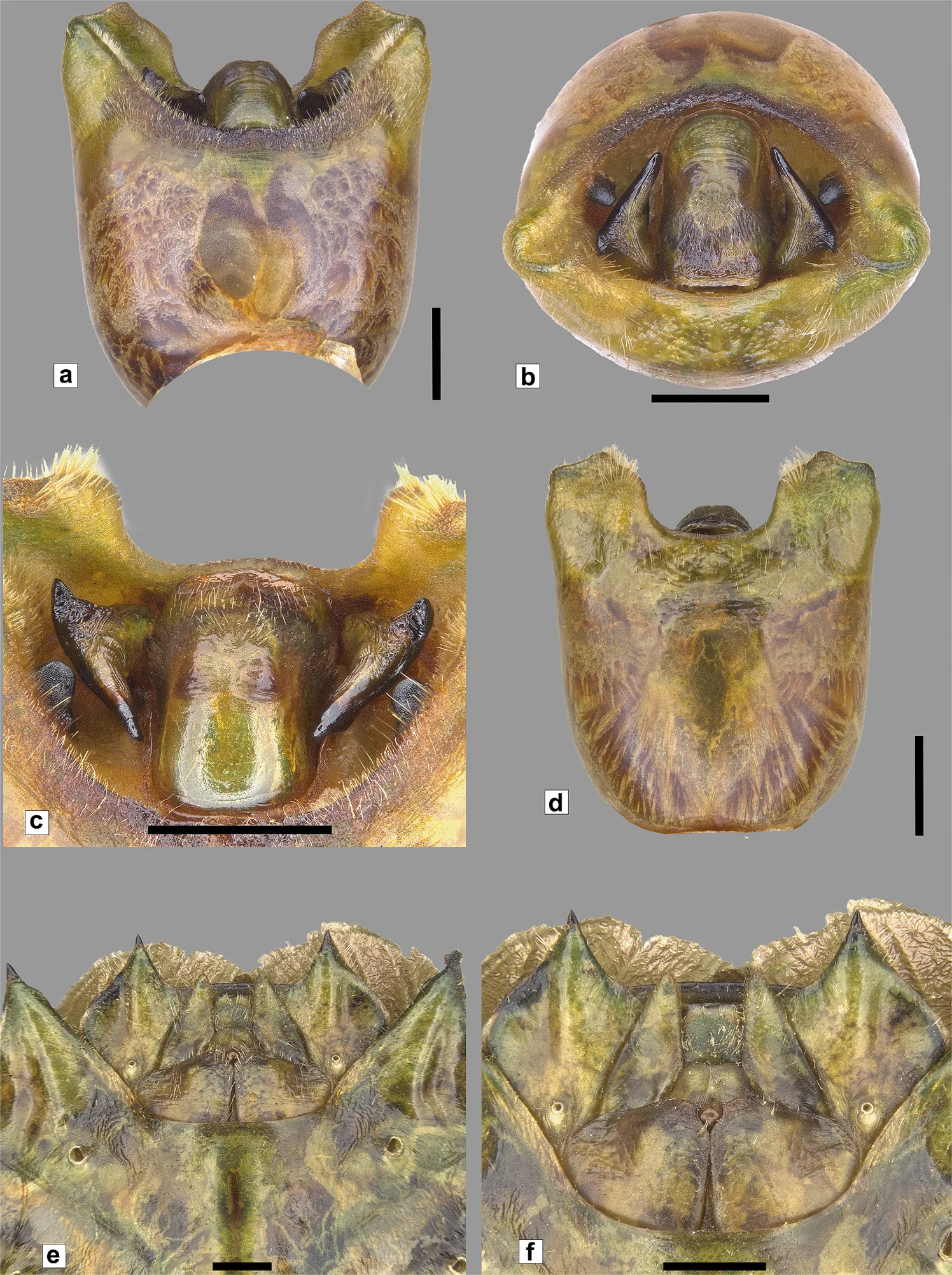
*Notochlora chlorosa* sp. nov. male genitalia (pygophore): **A**—dorsal view, **B**—posterior view, **C**—dorso-posterior view, **D**—ventral view. Female genitalia: **E**—ventral view, **F**—ventro-posterior view. Scale bars = 1 mm

**Fig. 6:**
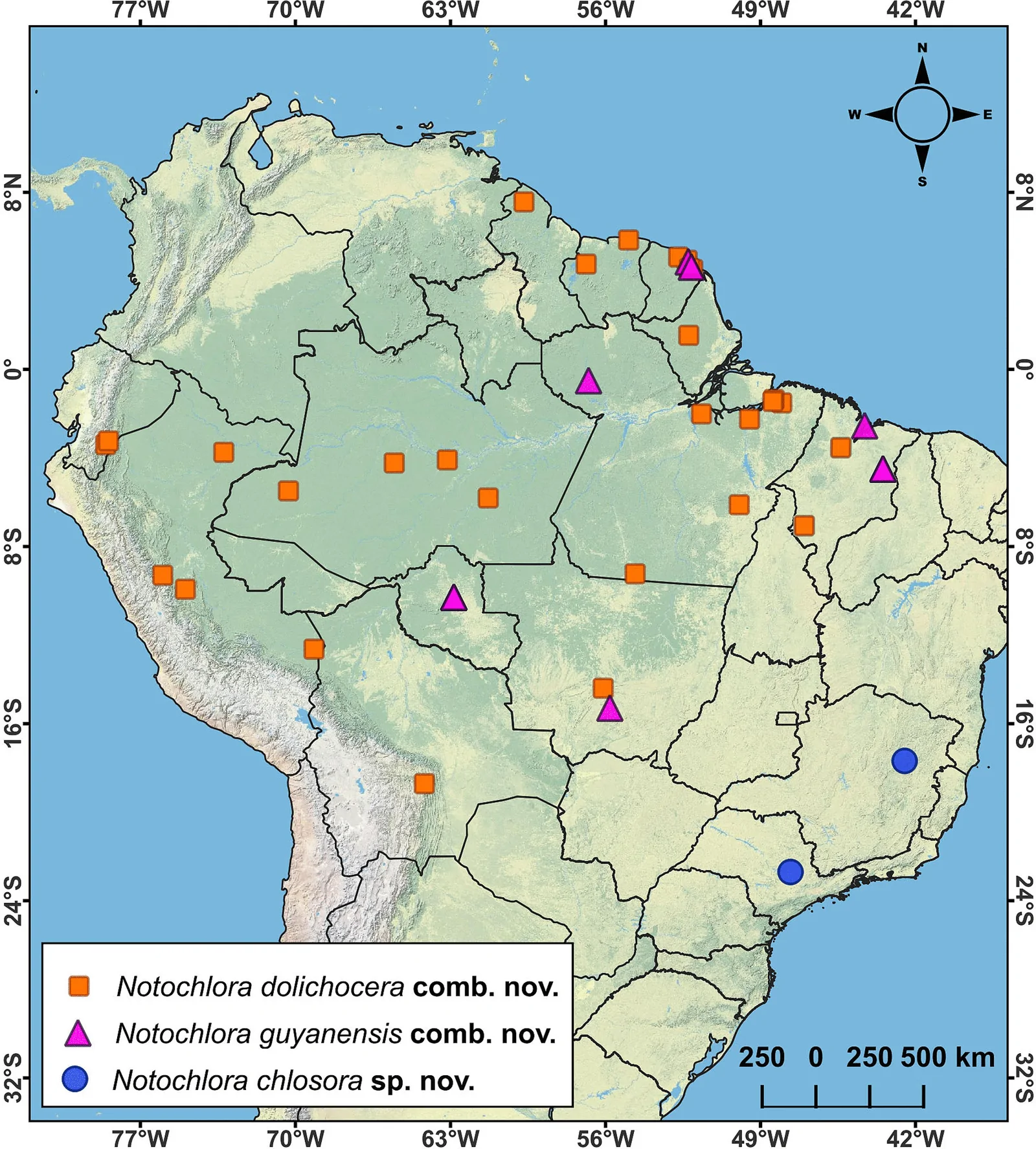
Distribution map of *Notochlora* species

**Fig. 7:**
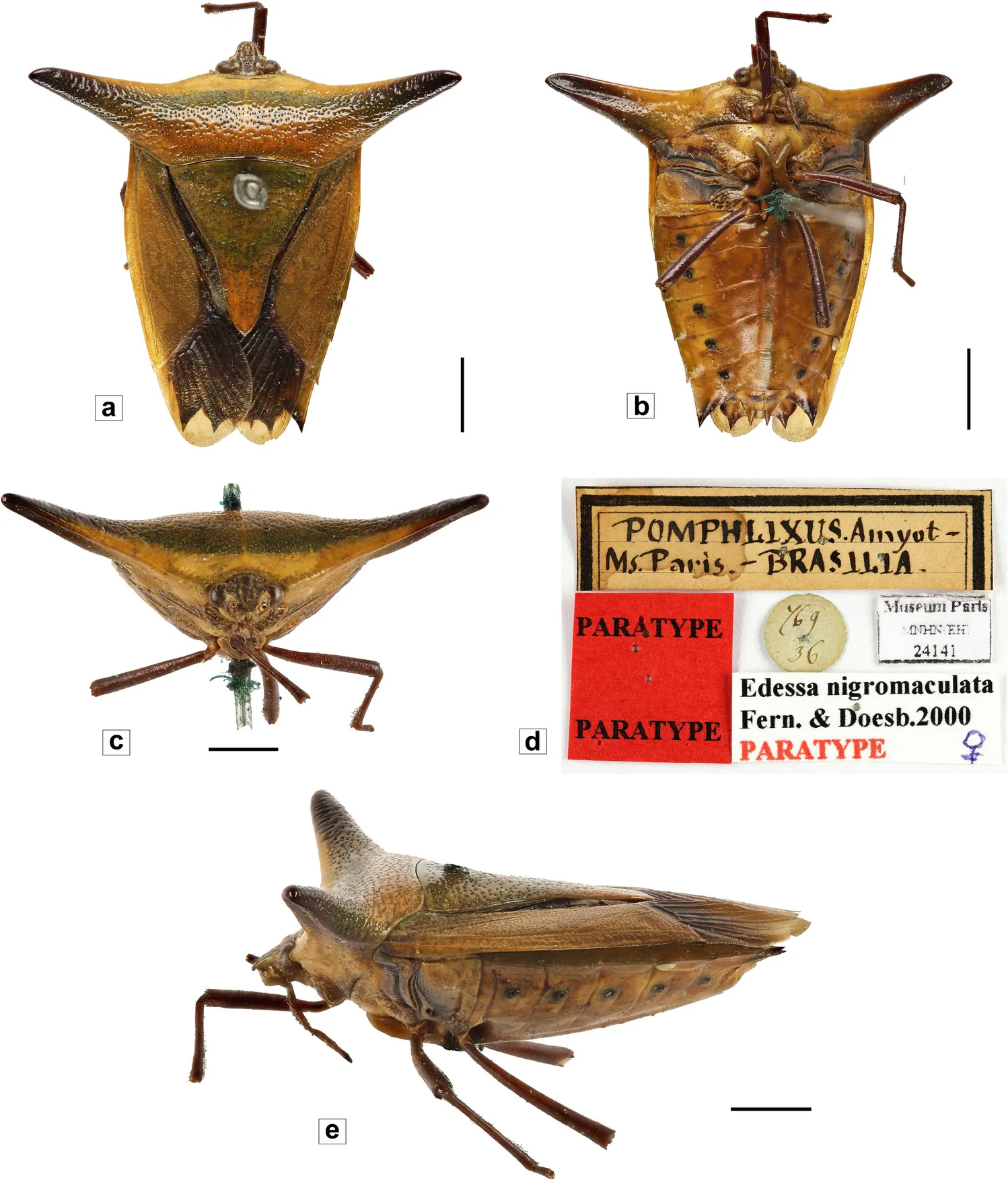
*Grammedessa nigromaculata*. Paratype. **A**—Dorsal view, **B**—ventral view, **C**—frontal view, **D**—specimen labels, **E**—lateral view. Scale bars = 5 mm

***Notochlora* gen. nov. Cunha, Robert and Fernandes** (Figs. 1, 2, 3, 4, 5, 6, and 7). ZooBank: urn:lsid:zoobank.org:act:C63F6EED-0C63-44E3-9130-6EE62B892DA6

**Type species:** *Cimex dolichocerus* Lichtenstein 1795.

***Etymology.*** The name of the new genus refers to the species' completely green dorsal coloration. Gr. *Notos,* back, Gr. *chloros*, (green), genus feminine.

**Definition**. Antennae reddish (Fig. 1). Humeral angles spiniform, well developed (about twice as long as it is wide) (Fig. 1). Connexivum homogeneous green or green with a middle yellow spot (Fig. 1a, c). Metasternal process shallow or deeply excavated anteriorly (Fig. 2). *Male genitalia*. Superior process of the genital cup is laminar, dark, and formed by two parts, with the dorsal part larger than the ventral part (Figs. 3c, 4c, and 5c). Ventral rim with excavated area and swollen (Figs. 3b–c, 4b–c, and 5b–c).

**Description.** Large (19.4–27.4 mm). Dorsal surface green, densely punctured (Fig. 1a, c, e); punctures shallow and concolor. Ventral surface yellow-greenish or green with transversal dark green stripes (Fig. 1b, d, f).

*Head.* Dorsal surface wider than long. Mandibular plates with or without yellow margin; apices bent ventrally and rounded; longer and contiguous in front of the clypeus. Bucculae subrectangular. Rostrum short, reaching the middle of the mesosternum (Fig. 1d, f); first segment surpassing bucculae, second segment a little bit shorter than third and fourth together.

*Thorax.* Pronotum green (Fig. 1a, c, and e). Anterior angle of the pronotum with a small tooth. Anterolateral margin of the pronotum curved, margin with shallow furrows, scars unpunctured. Humeral angles laterally or anterodorsally projected (Fig. 1a, c, and e); basal half punctured, posterior half unpunctured; posterior margin with shallow furrows, apices darker than base. Scutellum green, densely punctured, punctures concolor (Fig. 1a, c, and e); anterior half with large punctures, lateral and posterior areas with small punctures; apex acute (Fig. 1a, c, and e) and unpunctured. Corium green, densely punctured; hemelytral membrane castaneous with metallic sheen (Fig. 1a, c, and e). Propleurum distal half punctured with shallow and concolor punctures (Fig. 1b, d, and f). Metasternal process smooth; anterior arms with apex expanded laterally, harboring the fourth or third segment rostral (Fig. 2). Evaporatorium concolorous wrinkled; peritreme ruga-like, reaching ½ or 2/3 of the distance between the ostiole of the scent gland and the margin of the metapleuron. Legs green or yellow (Fig. 1). Femur with distal dorsal and lateral margin with small black lateral teeth (Fig. 1b, d, and f).

*Abdomen.* Dorsal surface green. Connexivum well-exposed, totally green or green with a middle yellow area, anterior and posterior areas excavated and punctured (Fig. 1a, c, and e). Urosternites II–VI with a small apical black spine (Fig. 1). Segment VII developed posteriorly, but not reaching the level of laterotergite VIII. Trichobothria in line with spiracles or one in line and the other lateral.

*Male genitalia*. Pygophore subrectangular (Figs. 3a, 4a, and 5a). Dorsal rim concave (Figs. 3a, 4a, and 5a), slightly rugose, setulose. Posterolateral angles not developed (Figs. 3a, 4a, and 5a). Superior process of the genital cup with dorsal part bigger than ventral part (Figs. 3c, 4c, and 5c). Paramere clavate (Fig. 3b) or developed anterodorsally, formed by two lobes, with the distal margin dark (Figs. 4b and 5b). Proctiger subcylindrical, slightly excavated laterally, posterior face ogival (Figs. 3b, 4b, and 5b). Ventral rim excavated medially; expansions with a tuft of setae (Figs. 3d, 4d, and 5d).

*Female genitalia.* Valvifer VIII convex (Figs. 3e, 4e, and 5e); posterior margin arched (Figs. 3f, 4f, and 5f); lateral slightly excavated. Valvifers IX convex (Figs. 3f, 4f, and 5f), with a median keel, surface with long setae. Laterotergite VIII convex, with a distal black, short spine, projected beyond the apex of segment VII (Figs. 3e, 4e, and 5e). Laterotergite IX trapezoidal with slightly excavated base, spiniform distal projection above the level of mediotergite VIII and almost at the same level as abdominal segment VII. (Figs. 3e, 3f, 4e, 4f, 5e, and 5f).

**Diagnosis.** *Notochlora* differs from other genera by reddish antennae, and green dorsal surface (this character is shared with *Pantochlora* Stål, 1870, but *Notochlora* has humeral angles developed, and in *Pantochlora*, the humeral angle is slightly developed). In addition, *Notochlora* has a humeral angle spiniform while in *Edessa* (*Edessa*) this angle is distally globose. The spiniform angle in *Notochlora* is shared with *Doesburgedessa* Fernandes, 2010 and the subgenus *Dorypleura* of *Edessa*, but this new genus differs from the others by its larger size and the entirely green humeral angle, whereas in *Doesburgedessa* and *Edessa* (*Dorypleura*) the humeral angles bear a brown or black spot.

**Remarks.** The map presented here (Fig. 6) is a compilation of collection points taken from Fernandes and van Doesburg (2000) and from the material examined in this study. This compilation provides a better idea of the distribution of the species belonging to *Notochlora* gen. nov.

**Distribution** (Fig. 6). Suriname, Brazil, Ecuador, Guyana, French Guiana, Peru and Bolivia.

***Notochlora dolichocera* (**Lichtenstein, 1795**) comb. nov.**

(Figs. 1a–b, 2a, 3, and 6) ZooBank: urn:lsid:zoobank.org:act:D21EDFB1-7A8C-4C76-BBFC-D043250ED812

*Cimex dolichocerus* Lichtenstein (1975): 106, 385.

*Edessa dolichocera*; Burmeister (1835): 355; Blanchard (1840): 143; Walker (1868): 429; Stål (1872): 59; Lethierry and Severin (1893): 190; Kirkaldy (1909): 157; Fernandes and van Doesburg (2000): 307.

*Edessa ductor* Walker (1868): 430; Lethierry and Severin (1893): 190; Kirkaldy (1909): 157; Fernandes and van Doesburg (2000): 307 (Synonymized).

**Material examined*.*** 4 ♀ and 1 ♂, no data (UFPA); 1 ♀, 1948–1949 (NNH). **BRAZIL**. **Amapá:** 1 ♀, Serra Navio, IX. 1959, Bicellicul leg. (MZSP). **Amazonas:** 1 ♀, Coari, 04.XI.2008, Fernandes. J. A.M. e equipe leg. (UFPA); 1 ♂, same locality, Petrobras, estrada RUC 18, Rio Urucu, 2.III.2010, Fernandes J. A.M. e equipe leg. (UFPA), P1 – borda de mata, S04° 53′ 54,9″ W65° 19′ 16,4″, luz mista Hg e luz negra, (UFPA); **Maranhão:** 1 ♂, Fazenda 7 irmãos, 03.06.2008, F. Lima de Oliveira J. A. Rafael & M. Moraes, cols.; 1 ♀, Bom Jardim, REBIO—Res. Biol., Gurupi, 17–27.01.2010, F. Limeira de Oliveira, J. T. Câmara & M. B. Aguiar Neto, cols. **Pará:** 1 ♂ and 1 ♀, Ananindeua, IX. 2014, Nascimento D. A. leg. (UFPA); 1 ♂ and 1 ♀, Belém 20 m, I.1984, V. O. Becker leg. (UFPA); 1 ♂, 04.XI.2009, F. S. Carvalho Filho. leg. (UFPA); 1 ♀, same locality, Campus do Museu Paraense Emílio Goeldi—MPEG, 11.IV.2012 (UFPA); 1 ♀, Campus MPEG, 21.10.2019, Arthur; 1 ♂, Parauapebas, Carajás, 08.XI.2008, Campos. L. D. leg., (UFPA); 9 ♂ and 7 ♀, Melgaço, Flona Caxiuanã, Estação Científica Ferreira Penna, coleta manual noturna sob luz branca, XI.2003, Fernandes, J.A.M. leg. (UFPA); 1 ♂, Serra Cachimbo, III.2004, Ricetti. J. leg. (UFPA); **Mato Grosso**: 1 ♀, Sinop, BR 163 km 496, x.1975, O. Roppa (MNRJ); 1 ♂, Cláudia, 09.10.2010, Smideri E. E. C.; 1 ♀, 13.09.2010, Barreto M. R.; 1 ♀, 10.10.2010, Miranda R. M. **ECUADOR. Morona-Santiago:** 1 ♀, Gualaquiza, 1895–96; 1 ♀, San Carlos de Coca, 21.IV.1985, G. Couturier Rec. (RL). **FRENCH GUIANA.** 1 ♂, Route de Kaw, PK 40, VII.1984, Piêge lumineux, G. Tavakilian Rec. (RL); 1 ♀, Bonne 4, IV.1983, (RL).

**Measurements (*N* = 46).** Total length: 19.4–21.7; head length: 1.1–2; head width, 3–3,9; antennae: I: 1–1.9; II: 1.1–4; III: 1.5–4.5; IV: 3.8–5; V: 3–5; pronotal length: 2.8–4.8; pronotal width: 9.8–23.3; humeral angle length: 3.6–4.4; humeral angle width: 2–3.1; abdominal length: 10–12.6.

**Description.** *Head.* Jugae with yellow margin. Second antennal segment twice as long as the first, third segment smaller than the second, fourth segment longer than the third and subequal to fifth. *Thorax.* Metasternal process with anterior arms short, not reaching the level of the anterior margin of mesocoxae (Fig. 2a). Peritreme reaches 2/3 of the distance between the ostiole of the scent gland and the margin of the body. Legs yellow to red (Fig. 1b). *Abdomen.* Connexivum green with yellow median spot (Fig. 1a). Trichobothria lateral to the line of spiracles. *Male genitalia.* Dorsal rim of the pygophore swollen distally (Figs. 3a–b). Posterolateral angles truncate (Fig. 3a). Superior process of the genital cup with claw-shaped dorsal part and rounded ventral part (Fig. 3c). Lateral wall of the pygophore with a small area densely setulose. Paramere spatulate with anterior and posterior lobes rounded and slightly developed (Fig. 3b). Proctiger with a dense tuft of setae on each side. Ventral rim with shallow median excavation and large, swollen conical expansions, with the expansions curved downward (Fig. 3b, d). *Female genitalia* (Fig. 3e, f)*.* Valvifer VIII with a swollen, rounded distal area and contiguous mesial borders.

**Diagnosis.** This species can be identified by the anterior excavation of the metasternal process, shallow and not reaching the mesocoxae, while in other species, this anterior excavation is deep, reaching the mesocoxae. Pygophore has the anterior lobe of the paramere rounded and slightly developed (in other species, this lobe is triangular and well-developed); expansions of the ventral rim are large and conical (in other species, these expansions are slightly developed). Proctiger with a dense tuft of long setae on each side (in the other two species, setae are short and sparse). In females, the posterior margin of valvifer VIII is more rounded than in the other two species.

**Distribution** (Fig. 6)**.** Suriname. Brazil: Amapá, Pará and Mato Grosso. Ecuador: Morona Santiago. Guyana: Barima-Waini. French Guiana: Cayenne, Kourou. Peru: Loreto, Pasco, Huánuco and Madre De Dios. Bolivia: Santa Cruz.

***Notochlora guyanensis* (**Fernandes and van Doesburg, 2000**) comb. nov. **(Figs. 1c–d, 2b, 4, and 6) ZooBank: urn:lsid:zoobank.org:act:E8ACFF15-43B9-4306-B688-8D2018EDC409

*Edessa guyanensis* Fernandes and van Doesburg (2000): 309.

**Material examined*.* SURINAME.** 1 ♀, Calkoen (RMNH). **FRENCH GUIANA:** 1 ♂, Cayenne, Piton Rocheuse, Crique Armontabo, II. 1984, 4°51′37.31″N, 52°16′09.20″W; 1 ♂, Regina, route de Kaw, 05.XI.1983, 4°32′21.32″N, 52°8′12.98″W, piége luminose, G. Tavakilian leg. (RL); 1 ♀, same locality, G. Tavakilian leg. (RL); 1 ♂, same locality, route de Kaw, PK 40, VII.1984, G. Tavakilian leg. (RL); 1 ♂, same locality, route de Kaw, PK 42, G. Tavakilian leg. (RL). **BRAZIL. Maranhão:** 1 ♂, Caxias, Campus UEMA, Morro do Alecrim, 23–30.12.2008, F. Limeira de Oliveira, 1 ♀, São Luis, V.1975, 2°33′25.92″S, 44°14′45.99″W (FURG).

**Measurements (*N* = 8)**. Total length: 23–27.4; head length: 2.9–3; head width: 4–4.1; antennae: I: 1.2–1.5; II: 2.2–2.6; III: 2.1–2.5; IV: 5.5–5.8; V: 5.5–5.9; pronotal length: 6.5–7.6; pronotal width: 25–28.2; humeral angle length: 6.7–7.6; humeral angle width: 3.3–4,1; abdominal length: 12.3–13.

**Description. ***Head*. Jugae with yellow margin. Second and third antennal segments subequal and twice as long as the first segment, fourth segment twice as long as the third, and fifth segment about the same size as the fourth segment. *Thorax*. Metasternal process with anterior arms long, laterally expanded apex, deeply excavated, reaching the level of mesocoxae (Fig. 2b). Peritreme reaches half of the distance between the ostiole of the scent gland and the margin of the body. Legs green (Fig. 1c–d). *Abdomen*. Connexivum green or with yellow median spot. Trichobothria one in line with spiracles and the other lateral. *Male genitalia*. Dorsal rim of the pygophore with a lateral castaneous spot (Fig. 4a). Posterolateral angles rounded (Fig. 4a, d). Superior process of the genital cup with a tooth-shaped dorsal part and a tiny, rounded ventral part (Fig. 4c). Paramere hammer-shaped, with long, conical anterior lobe and small, slightly acuminate posterior lobe (Fig. 4b). Proctiger with sparse and short setae. Ventral rim with deep median excavation; swollen and slightly projected expansions; distally with a small conical projection covered by a tuft of long setae (Fig. 4a, d). *Female genitalia* (Fig. 4e–f)*.* Valvifer VIII with a medial ridge; contiguous and distally excavated mesial borders. Valvifer IX with a globose protuberance at the base.

**Diagnosis.** *Notochlora guyanensis* comb. nov. and *N. chlorosa* sp. nov. share robust humeral angles; metasternal process with long anterior arms; tooth-shaped superior process of the genital cup; well-developed and acuminate anterior lobe of the paramere; slightly developed expansion of the ventral rim, deep median excavation, and a distal conical projection. *Notochlora guyanensis* comb. nov. can be distinguished from *N. chlorosa* sp. nov. by the slightly acuminate and small posterior lobe of the paramere (in *N. chlorosa* sp. nov. the posterior lobe of the paramere is triangular and more developed), and by the small ventral part of the superior process of the genital cup being partially fused with the larger part (in *N. chlorosa* sp. nov., the small part is completely fused with the larger part).

**Distribution** (Fig. 6)**.** Suriname. French Guiana: Cayenna. Brazil: Pará, Maranhão, Rondônia and Mato Grosso.

***Notochlora chlorosa* sp. nov. Cunha, Robert and Fernandes **(Figs. 1e–f, 2c, 5, and 6). ZooBank: urn:lsid:zoobank. org:act:903436E2-AF6A-400C-B365-AAA003C7D9EA

***Etymology.*** Gr. *Chloros*, green; refers to the green coloration of the body.

**Material examined.** *Holotype male*: **BRAZIL. Minas Gerais:** 1 ♂, Ouro Fino, Capelinha, 22°18′08″S 46° 23′43″W, 855 m, coleta ativa, 27.III.2024, L.Rubim. leg. (ESALQ). *Paratypes*: **BRAZIL.** 1 ♀, no data, 19.V.2006, Silva. E. leg. (ESALQ). **São Paulo:** 1 ♂, Piracicaba, 27.IX.2019, Santos L. E. leg. (ESALQ); 1 ♂, same locality, 26.XI.2022, Martins M. T. leg. (UFPA); 1 ♂, same locality, Escola Superior de Agricultura “Luiz de Queiroz” – ESALQ/USP, 16.VII.2018, Nerastri L. P. leg. (MZSP).

**Measurements (N = 5).** Total length: 20.8–24.6; head length: 1.6–2.3; head width: 3.5–3.9; antennae: I: 1.3–1.4; II: 2.1–2.6; III: 1.8–2.2; IV: lost; V: lost; pronotal length: 5.4–6.7; pronotal width: 23.3–25; humeral angle length: 3.6; humeral angle width: 6.8 m–7.2; abdominal length: 10.5–15.6.

**Description**. *Head.* Jugae with concolor margin. Second antennal segment twice as long as the first segment and slightly longer than third, the fourth segment twice as long as the third. *Thorax.* Metasternal process with anterior arms long, laterally expanded apex, deep excavated, reaching the level of the mesocoxae (Fig. 2c). Peritrema reaches 2/3 of the distance between the ostiole of the scent gland and the margin of the body. Legs green (Fig. 1f). *Abdomen.* Connexivum green (Fig. 1e). Trichobothria one in line with spiracles and the other lateral. *Male genitalia.* Dorsal rim of the pygophore with a castaneous spot (Fig. 5A). Posterolateral angles of the pygophore rounded (Fig. 5a). Superior process of the genital cup tooth-shaped and swollen, with a small rounded basal projection (Fig. 5c). Paramere hammer-shaped, anterior lobe subtriangular and long, posterior lobe small and triangular (Fig. 5b). Proctiger with sparse and short setae. Ventral rim with deep median excavation; swollen and slightly projected expansions; distally with a small conical projection covered by a tuft of setae (Fig. 5a–b). *Female genitalia* (Fig. 5e–f)*.* Valvifer VIII with a large, tumid distal area; basally divergent and distally excavated mesial borders. Valvifer IX with a globose protuberance at the base.

**Diagnosis.** See diagnosis of *N. guyanensis* comb. nov. Both species are very similar, but *N. chlorosa* sp. nov. was collected in the Atlantic Forest and *N. guyanensis* comb. nov. was collected only in the Amazon rainforest.

**Distribution** (Fig. 6)**.** Brazil: São Paulo and Minas Gerais.

***Grammedessa nigromaculata *(**Fernandes and van Doesburg, 2000 **) comb. nov. **ZooBank: urn:lsid:zoobank.org:act:CE7717A7-3920-4387-8D6F-29C242DF5EF4

*Edessa nigromaculata* Fernandes and van Doesburg (2000): 312.

During the analysis of the *E. dolichocera*-group (Fernandes and van Doesburg 2000) we noticed that one of the species described as part of this group, *Edessa nigromaculata*, is actually part of *Grammedessa* Correia and Fernandes, 2016. The presence of black stripes and rows of punctures on the head, as well as well-developed and flat humeral angles are characteristics found only in *Grammedessa* (Correa and Fernandes, 2016, Da Silva and Fernandes 2016 ).

Additionally, this species is characterized by a transverse yellow band on the anterior part of the pronotum about reaching the apices of the humeral angles; humeral angles are twice longer than wide, and the spiracles of the abdominal sternites have black circular stripes (Fig. 7). Among the *Grammedessa* species, the one most similar to *G. nigromaculata* comb. nov. is *G. graciligramma* Da Silva and Fernandes, 2022 . Both species share the triangular posterior margin of the valvifer VIII, which projects distally to the level of the apices of the valvifer IX. However, in *G. nigromaculata* comb. nov. the sutural margins of the valvifers VIII are straight, while in *G. graciligramma* it is sinuous.

Considering the change made here, the genus *Grammedessa* now has 20 species, listed below (Table 1):

**Table 1:** List of all species of *Grammedessa*

Species	Distribution
*G. acalyptoprocta* Da Silva and Fernandes 2022	Brazil: Maranhão
*G. brasiliana* Correia and Fernandes, 2016	Brazil: Minas Gerais, São Paulo, Rio de Janeiro
*G. bugabensis* (Distant, 1890)	Panama: Bugaba, Barro Colorado. Costa Rica: Heredia, Limón, Puntarenas
*G. brunneotarsata* Correia and Fernandes, 2016	Venezuela: Amazonas. Brazil: Amazonas
*G. flavolimbata* Correia and Fernandes, 2016	Guyana. French Guiana: Roura
*G. fundicava* Da Silva and Fernandes 2022	Brazil: Maranhão, Mato Grosso
*G. graciligramma* Da Silva and Fernandes 2022	Brazil: Maranhão
*G. hamata* (Walker, 1868)	Peru: Madre De Dios. Brazil: Amazonas
*G. hypsolineata* Correia and Fernandes, 2016	Brazil: Pernambuco
*G. longispina* Da Silva and Fernandes 2022	Brazil: Pará
*G. matogrossensis* Correia and Fernandes, 2016	Brazil: Mato Grosso
*G. multicavata* Correia and Fernandes, 2016	Peru: Madre De Dios. Bolivia: Chapare
*G. nigricava* Da Silva and Fernandes 2022	French Guiana: Saint-Laurent-Du-Maroni. Sinnamary, Saül
*G. nigromaculata* (Fernandes and van Doesburg 2000)*	Brazil: Pará
*G. pallicornis* (Walker, 1868)	Brazil: Pará
*G. paraensis* Correia and Fernandes, 2016	Brazil: Pará
*G. polytreta* Correia and Fernandes, 2016	Bolivia: Buena Vista, Chapare. Brazil: Mato Grosso
*G. rorativentris* (Breddin, 1903)	Bolivia: La Paz. Brazil: Amazonas
*G. stichtocephala* Da Silva and Fernandes 2022	French Guiana: Régina. Suriname: Sipaliwini
*G. stillativentris* (Breddin, 1905)	Colombia. Venezuela

^*^Specie with new combination

## Discussion

After Mendonça et al (2023) revised and diagnosed the subgenus *Edessa*, it became possible to accurately identify the species that belong to the genus *Edessa*. Based on our morphological revision of the species assigned by Fernandes and van Doesburg (2000) to the *Edessa dolichocera* group, we concluded that these species do not belong to *Edessa*. Instead, they represent two distinct taxa: a new genus, described herein, and *Grammedessa* Correa and Fernandes, 2016. *Grammedessa* was recently erected for several species formerly placed in *Edessa*, together with newly described species. During the present study, we recognized that *Edessa nigromaculata* also belongs to *Grammedessa*, based on the diagnostic characters of the genus. Accordingly, we transfer this species to *Grammedessa*, resulting in the new combination *Grammedessa nigromaculata*.

The new genus was described based on body morphology, characterized by a set of easily distinguishable characters. *Notochlora* differs from *Edessa* by having spiniform humeral angles, whereas in *Edessa* these angles are distally globose. The spiniform humeral angle in *Notochlora* is shared with the subgenus *Dorypleura* of *Edessa*; however, the new genus differs from the latter by its larger body size and green humeral angles, whereas in the subgenus *Dorypleura,* the humeral angles are brown to black with a metallic sheen. *Notochlora* also differs from the subgenus *Aceratodes* of *Edessa* by having well-developed humeral angles, whereas in *Edessa* (*Aceratodes*) the humeral angles are weakly developed.

## Declarations

### Nomenclature

ZooBank Registration Number: Cunha: 55BD4DE0- 5D10-4795-920F-636E338E7142, Robert: 26D18572-23B6-4A26-8F31-93259237BE80, Gomes: 2D65E2D3-06BD-4590-A7C7-7CC3CFCA2917; Fernandes: 1A4F5105-70FA-4B6A-810D-1061489BB65D.
